# Prescribed Hospital Diet Influence on Dietary Intake of Hospitalised Patients: A Cross-Sectional Study

**DOI:** 10.3390/nu17020261

**Published:** 2025-01-12

**Authors:** Joana Gameiro, Raquel Oliveira, Ana Lúcia Baltazar, Clara Rocha, Marta Pereira, Diana Martins, João P. M. Lima, Fernando Mendes

**Affiliations:** 1 Coimbra Health School (ESTeSC), Polytechnic University of Coimbra, 3046-854 Coimbra, Portugal; joanagameiro23@gmail.com (J.G.); ana.santos@estesc.ipc.pt (A.L.B.); clarapr@estesc.ipc.pt (C.R.); diana.martins@estesc.ipc.pt (D.M.); joao.lima@estesc.ipc.pt (J.P.M.L.); 2 Unidade de Nutrição e Dietética, Unidade Local de Saúde da Região de Leiria, Rua das Olhalvas, 2414-016 Leiria, Portugal; raquel.oliveira@ulsrl.min-saude.pt (R.O.); marta.a.carvalho@ulsrl.min-saude.pt (M.P.); 3 ciTechCare—Center for Innovative Care and Health Technology, Health Innovation Hub|Politécnico de Leiria, Campus 5, Rua das Olhalvas, 2414-016 Leiria, Portugal; 4 Higher School of Health, University of Algarve|University of Algarve—Campus de Gambelas, 8005-139 Faro, Portugal; 5 CERNAS—Research Center for Natural Resources, Environment and Society, 3046-854 Coimbra, Portugal; 6 Department of Electrical and Computer Engineering, INESC Coimbra, Polo 2, 3030-290 Coimbra, Portugal; 7 H&TRC—Health & Technology Research Center, Coimbra Health School, Polytechnic University of Coimbra, 3045-043 Coimbra, Portugal; 8 Biophysics Institute of Faculty of Medicine, Coimbra Institute for Clinical and Biomedical Research (iCBR) Area of Environment Genetics and Oncobiology (CIMAGO), University of Coimbra, 3000-548 Coimbra, Portugal; 9 Center for Innovative Biomedicine and Biotechnology, University of Coimbra, 3000-548 Coimbra, Portugal; 10 SUScita—Research Group for Sustainability, Cities and Urban Intelligence, Polytechnical University of Coimbra, 3046-854 Coimbra, Portugal; 11 European Association for Professions in Biomedical Sciences, 1000 Brussels, Belgium

**Keywords:** food intake, malnutrition, hospitalised patients, visual estimation method

## Abstract

Background: The dietary intake of hospitalised patients is often compromised during hospitalisation, which can be a causal factor for hospital malnutrition. This is considered a public health problem worldwide and is associated with an increased risk of other complications. Objectives: Our objective was to determine the dietary intake of hospitalised individuals and if the prescribed diet influences it. Methods: Food intake data were collected from 299 lunches of patients admitted to a hospital, using the visual estimation method with a five-point scale. Three existing diets were considered, and the energy and macronutrient values of the meal were calculated. The minimum energy and protein requirements were also calculated. Results: The components of the tray with the highest intake were soup and dessert; no significant differences were found between the percentage intake of each element and the prescribed diet. More than 50% of the individuals did not meet their minimum energy requirements, and only 36.5% had a protein intake that was within the recommendations. Conclusions: Dietary intake is much lower than prescribed, and nutritionists need to act to reduce the prevalence of hospital malnutrition.

## 1. Introduction

According to the European Society for Clinical Nutrition and Metabolism (ESPEN), malnutrition can be described as “a state resulting from lack of intake or uptake of nutrition that leads to altered body composition (decreased fat-free mass) and body cell mass leading to diminished physical and mental function and impaired clinical outcome from disease”. Malnutrition can result from different factors, such as hunger, illness, or advanced ageing, alone or in combination [1].

The food intake of hospitalised patients is often compromised during hospitalisation, which can contribute to malnutrition. This low intake can be caused by factors related to the illness itself, such as loss of appetite, nausea, or fatigue; the hospital, for example, interruptions during meals, unknown meal times, absence for exams, or fasting; or even individual factors, such as the need for help from others and physical disabilities [2,3]. Age-related issues such as presbyphagia and edentulism can affect chewing ability and effective food breakdown, requiring food consistency modifications. Common chronic conditions such as diabetes, hypertension, cardiovascular diseases, and chronic obstructive pulmonary diseases negatively impact appetite, nutrient assimilation, and utilization. Additionally, acute infections including pneumonia and urinary tract infections can cause an increase in metabolism and energy requirements [4].

Hospital malnutrition is considered a public health problem worldwide. It is associated with more extended hospital stays, higher risks of complications, more frequent readmissions, higher mortality, and, consequently, higher healthcare costs that all affect the quality of life compared to adequately nourished patients [2,3,5].

In most situations, low-cost treatments based on dietary interventions are effective if malnutrition is diagnosed early by identifying nutritional risk [6]. In Portuguese National Health System hospitals, nutritional risk should be identified within the first 48 h of admission, using the Nutritional Risk Screening 2002 (NRS 2002) tool for adult patients and STRONGkids for paediatric patients.

When nutritional risk is identified, the patient is referred to the nutrition department, which assesses their nutritional status, establishes the nutritional diagnosis and intervention, and monitors the evolution of the patient’s nutritional status [1,7]. A survey carried out in 49 Portuguese public hospitals between January 2019 and December 2020 found that for the year 2020, of the 424,721 patient records, 27.1% were screened. Of these, one in four patients was nutritionally at risk (25.6%) [7].

In addition to the factors already mentioned, the hospital diet itself can increase the risk of malnutrition. Thus, the quality of the food the hospital provides plays an important role in the maintenance, recovery, and prevention of deterioration in the patient’s nutritional status. Therefore, to provide adequate nutrition to meet individual energy and nutritional needs, it is essential to prescribe an appropriate diet. In Portuguese public hospitals, general and light diets, paediatric diets, individual option diets (ovolactovegetarian and vegetarian), modified texture diets, and therapeutic diets are available [8]. It is common for hospital diets to be unappetizing to patients, either due to restrictions imposed by texture modifications, reduced salt content, or inadequate meal temperature. These factors can negatively affect palatability, food variety, texture, and visual appearance of the meal, leading to a decreased adherence and subsequently contributing to malnutrition [4,9].

Regarding food intake during hospitalisation, one study concluded that a low intake was observed in one in five patients and also found a reduction in intake in 27.7% of patients [2]. According to the literature, the components of the tray with the least waste and, therefore, the highest food intake are soup and dessert, while the main course has the lowest food intake [10,11,12,13].

Given the high prevalence of malnutrition in hospitals, this study aimed to determine the food intake of hospitalised individuals and see if the prescribed diet influenced it.

## 2. Materials and Methods

### 2.1. Type of Study

A cross-sectional observational study was conducted in patients hospitalised, where over a period of twenty days, between 31 October 2022 and 12 January 2023, data were collected on the dietary intake of patients admitted to a 30-bed internal medicine ward of a hospital in the centre of Portugal. A total of 299 lunches were assessed, with 127 from the soft diet (42.5%), 104 from the standard diet (34.8%), and 68 from the diet restricted to simple sugars and saturated fat (22.7%). The hospital’s internal medicine department, where the study was carried out, consists of four wards with 30 beds each, totalling 120 beds. In this ward, since 2018, there has been a predominance of female inpatients (53.1%) who have an average age of 76.1 years. The most prevalent diseases in the ward were those of the respiratory system (46.4%), followed by diseases of the circulatory system (21.8%) and genitourinary system (13.9%), with an average mortality rate of 15.5%.

The research methodologies were in accordance with the guidelines outlined in the Strengthening the Reporting of Observational Studies in Epidemiology (STROBE) [14,15].

### 2.2. Data Collection

The food weighing method is the gold standard for monitoring food intake; however, in a hospital environment, its use becomes difficult as it is disruptive to food service activities, costly, and time-consuming. The visual estimation method was suggested as an alternative [16]. According to this method, observers estimate the portion size for each food, considering the standard portions that were previously weighed, leading to an indication of the proportion of waste of the initial portion (e.g., 0%, 25%, 50%, 75%, and 100%) [10,16]. Studies have also been carried out in hospital settings using this method to determine patient food intake [17,18,19].

In the current study, the amount of each component of the tray eaten was classified on a five-point scale, namely 0%, 25%, 50%, 75%, and 100%, with the tray in question consisting of soup, protein (meat, fish, or eggs), side dish (potatoes, rice, or pasta), vegetables, and dessert (fruit). Bread consumption was not considered as it was only included in the standard diet. The principal investigator always performed the visual estimation; no personal data were collected from the patients.

The diets included were the standard hospital diet, the soft diet, and the diet restricted to simple sugars and saturated fat derived from the light diet (Table 1). These diets are prescribed more frequently than those in the department in question and are included in the Hospital Diet Manual [8].

Subsequently, using the categories described in the Hospital Diet Manual and the New Table of Nutritional Equivalents, each meal’s average energy and nutritional values (protein, carbohydrates, and lipids) were calculated, taking into account the estimated percentage of intake [8,20].

The average daily energy and protein requirements were also calculated using the values in the Hospital Diet Manual, which were 2046 kcal and 102.3 g, respectively. Calculating 30% of these values, since it is stipulated that lunch should satisfy 30 to 35% of daily energy needs, resulted in 613.8 kcal and 30.69 g as the recommended lunch intake for hospitalised patients. This manual does not consider the increase in energy needs. However, these may be increased in situations of illness since, during hospitalisation, there is a decrease in physical activity levels. These needs are also calculated for the adult population since, although there are differences in the energy needs of the elderly, there is no justification for developing specific diets for this population group [8].

Subsequently, to calculate the minimum requirements needed to guarantee basal metabolism, the average basal metabolism rate values for men and women were calculated, resulting in 1364 kcal. From this figure, 30% was calculated, obtaining 409.2 kcal for lunch. Concerning protein intake, the EFSA (European Food Safety Authority) recommends a PRI (Population Reference Intake) value of 0.83 g/kg of weight/day, considering a protein intake that does not exceed more than 2 times the PRI value to be safe. Thus, using the average weight values described in the Hospital Diet Manual and calculating the average for the values for men and women, 48.89 g/day was reached. After calculating 30%, 14.67 g of protein was obtained [8,21].

### 2.3. Statistical Analysis

The food intake data and the calculations of energy and nutritional values were recorded in Microsoft Office Excel software and then transferred to IBM SPSS Statistics software, version 28.0, for statistical analysis.

Quantitative variables were expressed as means ± standard deviation and qualitative variables as frequencies. The non-parametric Kruksal–Wallis test was used to study the existence of significant differences between the food intake of the different components of the tray and the prescribed diet. To see if there was a statistically significant association between the adequacy of the calculated minimum energy and protein requirements and the prescribed diet, Pearson’s Chi-Square non-parametric statistical test was used. The statistical significance criterion was a *p*-value < 0.05 for a 95% confidence interval.

### 2.4. Ethical Approval

This study was approved by the hospital’s Nutrition Department, Internal Medicine Department, Ethics Commission (no. 71/CECHL/2023) from 9 August 2023, and the Administration Council of the Centro Hospitalar de Leiria, where the study was conducted.

The study respects the principles of the Declaration of Helsinki, ensuring maximum protection and confidentiality of the data obtained from the participants.

## 3. Results

**Figure 1:**
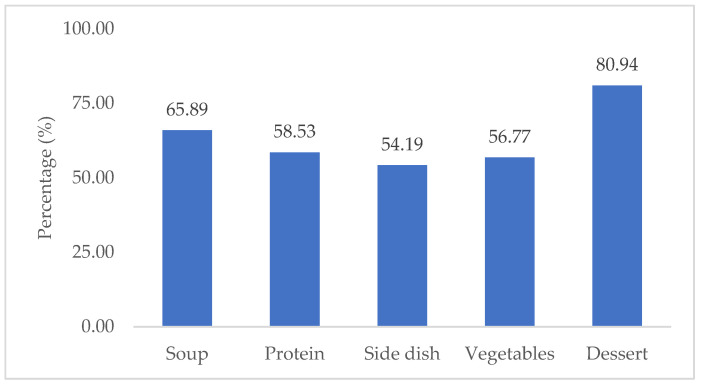
Mean percentage of total intake of the tray components.

Concerning the estimated percentages of food intake, it can be seen in Figure 1 that the components of the tray with the highest intake are dessert (80.94%), followed by soup (65.89%), and in the main course, the side dish has the lowest intake (54.19%), followed by vegetables (56.77%), and protein has the highest percentage of intake (58.53%).

**Figure 2:**
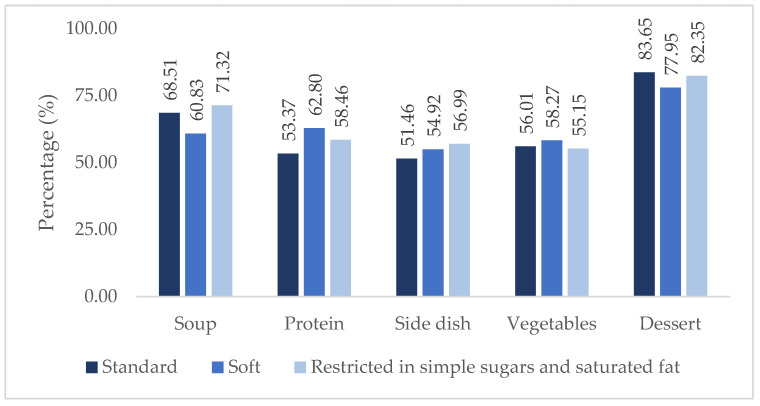
Mean percentage intake of tray components per diet.

Figure 2 shows the percentages of intake of the different components of the tray by diet. It was found that soup had a lower intake on the soft diet and a higher intake on the diet restricted in simple sugars and saturated fat (60.83% and 71.32%, respectively). Protein had a lower intake on the standard diet (53.37%) and a higher intake on the soft diet (62.80%), and there was a lower intake of the side dish in the standard diet (51.46%) and a higher intake on the diet restricted in simple sugars and saturated fat (56.99%). In vegetables, the lowest percentage of intake was on the diet restricted in simple sugars and saturated fat and the highest intake was on the soft diet (55.15% and 58.27%, respectively). Finally, in dessert, the highest intake was on the standard diet and the lowest intake was on the soft diet (83.65% and 77.95%, respectively). However, no statistically significant differences (*p* ≥ 0.05) existed for any of the components.

The calculated average energy and protein values of all the meal components of the different diets are shown in Table 2, as well as the total averages of the meals of each diet, including both the energy and protein consumed and the total intake provided by the hospital. In total, disregarding the different diets, there was an average consumption of 324.63 ± 164.02 kcal and 20.21 ± 11.57 g of protein.

As shown in Table 3, for the energy needs, regardless of diet, more than half of the individuals had an intake below the 409.2 kcal needed to meet their minimum needs (57.9%). Regarding total protein intake, only 36.5% were within the recommendations (14.64 g to 30.69 g), 36.5% were below, and 27% were above the protein recommendations.

All the diets showed a percentage of more than 50% inadequate energy requirements, with 62.5%, 55.1%, and 55.9% for the standard, soft, and simple sugar and saturated fat-restricted diets, respectively. There was no statistically significant association between the adequacy of energy requirements and the prescribed diet (*p* = 0.492).

In terms of protein intake, it was found that less than 40% of individuals on all diets had an intake within the recommended range of 14.64 g to 30.69 g. It was also found that 42.3% of individuals on the standard diet had an intake below the recommended range, as did 33.1% and 33.8% of individuals on the soft diet and the diet restricted in simple sugars and saturated fat, respectively. There was also no statistically significant association between the adequacy of protein requirements and the prescribed diet (*p* = 0.256).

## 4. Discussion

The results show a higher intake of dessert in all diets and a higher intake of soup than the main course in the standard and simple sugar- and saturated fat-restricted diets. There was a higher protein intake than soup on the soft diet, which could be explained by the fact that patients on this diet are more debilitated and more likely to need help from other people during the meal, and they prioritise the consumption of meat, fish, and eggs. The percentage of side dishes eaten on the standard diet was lower than on the other diets, which could be explained by the consumption of bread, which was not considered in this study. On the other hand, there was a lower percentage of vegetable consumption in the diet restricted to simple sugars and saturated fat since more vegetables are served in this diet.

The lower intake of the main course compared to the consumption of soup and fruit is in line with previous conclusions by other researchers. In particular, in a study carried out in Portugal by Dias-Ferreira et al., it was also found that the main course had a lower intake, as it had a higher percentage of food waste (FW). At the same time, dessert and soup were well accepted, with a waste of 10% and 12%, respectively [10]. In the study by Dias and García et al., dessert had a higher intake, while vegetables were less accepted by the patients in a hospital in Spain [11]. In a study by Gomes et al., the average percentage of FW during lunch for soup was 32.4 ± 9.2%. For the main meal, the side dish had the highest FW value (58.6%), followed by vegetables with approximately 57.6% and, finally, protein (45.2%) with the lowest FW value [12].

A study in three hospitals in Italy found that 41.6% of the food served at lunch and dinner was wasted. The wastage percentages were lowest for fruit (35.2%), and for the main course, vegetables had the highest wastage (55.0%). However, the component with the least waste was the side dish (38.5%), followed by protein (39.7%) [13]. In a study carried out in Germany, food intake was assessed on one day, as well as the week before hospitalisation. It was found that in the week before hospitalisation, 31.0% of patients reported a reduction in food intake; on the day of the study, 49.5% ate only half or less of the meal served, and 27.7% of patients had reduced their intake only on the day of the study, but not in the week before admission. The researchers therefore concluded that a low food intake was observed in one in five patients [2].

Possible causes of low food intake during hospitalisation have already been described in several studies, the most common reasons being lack of appetite, nausea, fatigue and pain related to the illness, the quality of the meals served since several studies have identified lack of salt and inadequate meal temperature as factors for low intake, the carrying out of examinations or surgeries, and the need for assistance during meals, which is not always provided. Personal factors can also contribute to low intake, such as physical disabilities and being in unsuitable positions to eat, for example, lying in bed [22,23,24,25,26].

This study’s results also show no significant differences between food intake and the prescribed diet, which is in line with a 2020 study where there were no significant differences between food waste and the prescribed diet [12]. This also agrees with the study by Rattray et al., which found that only patients on oral fluid diets had significantly lower energy and nutrient intakes than patients on other diets [27]. On the other hand, Kandiah et al. conducted a study in which a visual plate waste method during lunch was analysed for four consecutive days. It was concluded that diabetic diets (diets restricted to carbohydrates) and altered consistency diets significantly affected plate waste. In particular, patients receiving a diabetic diet showed less FW. On the contrary, an increase in FW was observed in patients receiving altered consistency diets. The other diets did not significantly affect FW [28].

The economic impact of food waste can be found in different studies around the world. A study at an acute care hospital in Portugal verified that, on average, each patient throws away 953 g of food each day, which equates to almost EUR 1.5 million per year of food waste [10]. Another study performed in Italy confirmed that the adoption of a more varied hospital diet, like the modified homogenized diet, which consists of foods formulated with natural ingredients that are of top quality and from a controlled supply chain and that have a creamy consistency, density, and high lubricating action for safe swallowing, was more effective and favoured by patients compared to a homogenised standard diet. This resulted in less food waste and in a lower daily cost for the hospital [9].

A systematic review identified studies that evaluated several strategies with the aim of increasing patient food intake, improving their health status, reducing food waste, and consequently lowering the hospital’s associated costs, including food service systems like catering and room service, protected meals and voluntary food assistance, better food presentation, nutrition counselling and education, and plant-based protein meals [25].

This study calculated an average consumption of 324.63 ± 164.02 kcal and 20.21 ± 11.57 g of protein at lunch. These figures are similar to those estimated by Bjornsdottir et al., namely an energy intake of 319 ± 133 kcal and a protein intake of 18.1 ± 7.7 g at lunch in a hospital setting in Iceland [29]. Also, in the study by Weijzen et al., published in 2019, the protein intake at lunch was 22 ± 11 g for male patients and 16 ± 7.0 g for female patients [30]. Considering low food intake as a risk factor for malnutrition, the minimum amount of energy that should be ingested during lunch was subsequently calculated to guarantee the individuals’ basal metabolism rate. The value obtained was 409.2 kcal; the results show that 57.9% of individuals do not reach this value.

Regarding protein, the recommended intake range of 14.67 to 30.69 g at lunchtime was calculated according to EFSA recommendations. It was found that only 36.5% of individuals had an intake in this range, with 36.5% having a lower intake and 27.1% having a higher intake. As only lunchtime intake was considered in this study and no patient data were collected, the possible intake of oral nutritional supplements needed to be considered. Therefore, although 36.5% of the individuals had an intake below the minimum recommended protein value at lunchtime, this number may be lower, considering the patients’ entire dietary day. Concerning the individuals with a higher-than-recommended intake (27.1%), this can be explained by the fact that the Hospital Diet Manual considered the maximum limit of the recommendations defined by the EFSA since it was the value that most closely matched the dietary habits of the Portuguese population, according to the National Dietary and Physical Activity Survey, 2015–2016 [8,31].

Simzari et al. carried out a study in which they showed that only 11.7% of patients met their daily energy needs (2030.3 ± 409.03 kcal), and 9.2% met less than 25% of their needs. As for protein, 15.8% met less than 25% of the recommended daily requirement (76.13 ± 15.33 g). This study also concluded that hospital malnutrition is highly prevalent, along with a high rate of food waste and nutritional risk [32]. Rosenberger et al.’s study of 330 patients found that only 10.6% of patients ingested more than 0.8 g/kg of weight/day of protein and that 21.2% met the minimum daily energy recommendations (considering 20 kcal/kg of weight/day) [33]. The average protein intake observed in the study by Weijzen et al. carried out in 2020 was 0.65 g/kg of weight/day, with only 35% of patients meeting the recommendations of 0.8 g of protein/kg of weight/day [34].

The study by Dupertuis et al. found that the total food intake of 43% of hospitalised patients was below their minimum nutritional requirements, which are necessary to maintain basal metabolism (1293 ± 246 kcal/day and 51 ± 10 g of protein/day) [35].

Compared to the literature consulted, it can be seen that in the present study, there was greater adequacy of energy and protein needs; however, the percentage of inadequacy is considerably high since there was a low food intake, which is one of the factors for the high prevalence of malnutrition in hospital settings. Food intake and nutritional screening are fundamental to healthcare and should be carried out regularly on all hospitalised patients. A multidisciplinary approach is both clinically and cost-effective in the management of malnutrition. It is, therefore, essential to match the number of nutritionists to the number of hospital beds and train other healthcare professionals in hospital malnutrition, particularly nurses, who have a crucial role in the identification, prevention, and treatment of malnutrition, along with assistants, since they have a significant amount of daily contact with patients, including giving assistance during mealtimes [6,36].

This study has several limitations, including that although the visual estimation method has been validated, it is biased towards a higher percentage of error than other methods for monitoring food intake, such as weighing food. Averages were calculated to calculate the energy and protein values of the meals, taking into account the categories of the Hospital Diet Manual and the New Table of Nutritional Equivalents. The average energy and nutritional requirements calculated for a healthy adult population were used despite the study being conducted in a hospital setting, in a service with a high prevalence of elderly people. The use of oral nutritional supplements was not considered. Finally, due to the limited number of articles on food intake, food waste studies were included.

## 5. Conclusions

The amount of food eaten directly impacts the clinical evolution of hospitalised patients since low food intake is one of the causal factors of malnutrition. More studies should, therefore, be carried out on food intake, and, in particular, the constraints on food intake should be assessed with the patient.

It was concluded that the prescribed diet in a hospital environment does not influence actual patient food intake. However, large-scale studies are needed to verify these results, also using the visual estimation method or, if possible, weighing food, in order to obtain more accurate results. The study was only carried out in one hospital department; therefore, it would be interesting to carry out futures studies in different wards, where patients have other more prevalent illnesses, and to evaluate food intake from different hospital diets.

It was also concluded that food intake is much lower than prescribed, with more than half of the patients not meeting their minimum energy and protein requirements. Therefore, nutritionists must ensure that patient diets are appropriate, that nutritional supplements are recommended, and that the nutritional status and nutritional needs of hospitalised patients are monitored daily to help reduce the prevalence of malnutrition in hospitals.

## Figures and Tables

**Table 1:** Diet characteristics according to the Hospital Diet Manual [8].

Diet	Diet Characteristics
Standard hospital diet	A diet that follows the principles of a healthy, normoenergetic diet adjusted to the nutritional needs of the adult population. It is intended for patients who do not have specific nutritional or dietary needs.
Soft diet	A modified texture diet with food in a soft consistency that allows easy chewing and/or swallowing, reducing the risk of choking and aspiration. The food can have a smooth and chopped consistency (IDDSI consistency level 6), when it is naturally soft or cooked or cut to change its texture, or a ground and moist consistency (IDDSI consistency level 5) when the food is ground and moist making it easily mashable with a fork, and the lumps are smooth and round.
Diet restricted in simple sugars and saturated fat	A diet restricting fast-absorbing carbohydrates and saturated fat, with the aims of glycaemic control, weight loss, lipid profile control, and overall metabolic control. It is suitable for patients with diabetes, hyperglycaemia associated with corticosteroid therapy, obesity, dyslipidaemia, and metabolic syndrome.

**Table 2:** Calculated average energy and protein values of the meal components of the different diets.

Diet	Meal Component	Energy (kcal)		Protein (g)	
		Consumed (M ± SD)	Total Intake	Consumed (M ± SD)	Total Intake
Standard	Soup	92.78 ± 56.41	135.42	4.32 ± 2.63	6.31
	Protein	78.56 ± 61.98	147.21	10.37 ± 8.18	19.43
	Side dish	72.97 ± 60.69	144.45	2.97 ± 2.47	5.82
	Vegetables	10.21 ± 7.89	18.23	0.77 ± 0.60	1.38
	Dessert	66.66 ± 28.69	79.69	1.57 ± 0.68	1.88
	Total average	304.53 ± 166.25	527.55	18.66 ± 11.92	34.82
Soft	Soup	82.37 ± 52.34	135.42	3.84 ± 2.44	6.31
	Protein	92.44 ± 61.49	147.21	12.20 ± 8.12	19.43
	Side dish	80.73 ± 62.81	147.00	2.20 ± 1.71	4.00
	Vegetables	10.62 ± 7.40	18.23	0.81 ± 0.56	1.38
	Dessert	62.12 ± 29.55	79.69	1.47 ± 0.70	1.88
	Total average	337.54 ± 165.42	527.55	21.41 ±11.60	34.82
Restricted in simple sugars and saturated fat	Soup	75.70 ± 38.70	106.14	3.25 ± 1.66	4.55
	Protein	86.05 ± 61.52	147.21	11.36 ± 8.12	19.43
	Side dish	83.77 ± 63.45	147.00	2.28 ± 1.73	4.00
	Vegetables	20.11 ± 16.34	36.47	1.52 ± 1.24	2.76
	Dessert	65.63 ± 29.62	79.69	1.55 ± 0.70	1.88
	Total average	331.26 ±157.24	516.51	20.32 ± 10.83	34.82

Abbreviations Legend: M—mean; SD—standard deviation.

**Table 3:** Adequacy of energy and protein requirements in the different diets.

Diet	Energy Requirements n (%)			Protein Requirements n (%)			
	<409.2 kcal	≥409.2 kcal	*p*	<14.64 g	14.64–30.69 g	>30.69 g	*p*
Standard	65 (62.5%)	39 (37.5%)	0.492	44 (42.3%)	40 (38.5%)	20 (19.2%)	0.256
Soft	70 (55.1%)	57 (44.9%)		42 (33.1%)	45 (35.4%)	40 (31.5%)	
Restricted in simple sugars and saturated fat	38 (55.9%)	30 (44.1%)		23 (33.8%)	24 (35.3%)	21 (30.9%)	
Total	173 (57.9%)	126 (42.1%)		109 (36.5%)	109 (36.5%)	81 (27%)	

Abbreviations Legend: n—number of meals; %—percentage.

## Data Availability

Data are contained within the article.
